# Heavy Metal Contamination of Ground Water from an Unlined Landfill in Bulawayo, Zimbabwe

**DOI:** 10.5696/2156-9614-7.15.18

**Published:** 2017-09-07

**Authors:** Charles Teta, Tapiwa Hikwa

**Affiliations:** Department of Environmental Science and Health, National University of Science and Technology, Bulawayo, Zimbabwe

**Keywords:** solid waste, landfill, leachate, heavy metals, groundwater

## Abstract

**Background.:**

Developing countries such as Zimbabwe deal with challenges in solid waste management such as insufficient waste recycling, hazardous wastes that are not separated for safe disposal, and landfills that are not properly engineered to prevent groundwater pollution. For these reasons, landfills in developing countries pose serious environmental and public health hazards.

**Objectives.:**

The objective of this study was to assess heavy metal release and groundwater pollution from an unlined landfill in Bulawayo, Zimbabwe. The purpose of the study was to explore environmental and public health risks posed by improperly managed landfills in developing countries.

**Methods.:**

We assessed levels of metal release from Richmond landfill in the city of Bulawayo, Zimbabwe by measuring lead, cadmium, chromium and copper levels in landfill soil, leachate and plants. We also monitored metal levels in groundwater from boreholes located in a residential area in the vicinity and downgradient of the landfill within a range of 800–2135 m. Soil was characterized at the landfill to assess potential sources of heavy metals.

**Results.:**

All metals that were assessed were present in landfill soil and in leachate. There was high metal accumulation in weeds that were growing at the landfill, indicating mobility and bioavailability of the metals. Groundwater from nearby boreholes had high levels of lead (Pb) and cadmium (Cd) which were negatively correlated to distance from the landfill (p<0.01), indicating contamination from the landfill. The Pb and Cd levels exceeded World Health Organization standards for drinking water quality, posing health hazards to the communities who rely on the water. Solid waste at the landfill consisted of soft plastics (33%), hard plastics (18.6%), metals (3%), paper (8%), electronic waste (0.8%), organics (15.3%) and various other types (21.3%).

**Discussion.:**

A combination of factors may be attributed to groundwater contamination. These include the co-disposal of metallic and electronic wastes at the landfill, lack of membrane lining at the landfill, inadequate leachate management and the porous geo-physical characteristics of the sub-surface at the landfill site.

**Conclusions.:**

Our study highlights adverse environmental and public health consequences of co-disposal of metals and electronic wastes at improperly engineered municipal landfills. This is a ‘wake-up’ call for policy makers in developing countries to improve solid waste management.

## Introduction

Landfills are used worldwide as a cost-effective method of solid waste disposal. However, there is concern about their adverse effects on the environment, particularly as sources of hazardous pollutants to groundwater. Due to the diverse nature of solid wastes at landfills, a variety of toxic chemicals are commonly reported in landfill leachates and pose contamination risks to the surrounding sub-surface water.^1^

As rainwater soaks through layers of solid waste in a landfill, chemical, biological and physical processes cause the leaching of hazardous chemicals from various waste materials to form hazardous leachate.^2^,^3^ Some of the toxic pollutants that are known to leach from landfills include heavy metals, polybrominated diphenyl ethers, polychlorinated biphenyls, bisphenol A, and other potentially toxic organic compounds.^4–10^ The extent of this risk depends on the toxicity of pollutants, levels of pollutants in leachate, the physical characteristics of underlying geological strata (soil type and permeability), water table depth and the flow of groundwater.^11^

Solid wastes such as scrap metals, metallic devices, batteries and electronic wastes found in landfills are some of the sources of heavy metals in landfill leachate. Non-essential metals such as lead, cadmium, chromium and mercury are highly toxic, even at very low concentrations.^12–14^ Moreover, these metals have been shown to accumulate in plant and animal tissues, therefore even low exposure concentrations can bioaccumulate during prolonged exposures to cause toxicity.^15–17^ The metals lead (Pb), cadmium (Cd) and chromium (Cr) have serious health effects in humans.^18^ Lead and Cd are linked to neurological, kidney and brain damage.^19^ Children are particularly susceptible to Pb poisoning because they absorb up to 5 times more Pb than adults.^19^,^20^

In an effort to reduce the toxicity of landfill leachates, various leachate treatment methods have been developed, but these methods are both complicated and costly, especially for developing countries.^21–23^ In modern landfills, membrane lining of landfill sites and leachate ponds have been preferred alternatives to minimize contamination of groundwater, although breakthroughs into the subsurface may still occur. Heavy metals are among the most difficult pollutants to clean from the environment due to their persistence, and polluted sites present long term environmental challenges.^24^ Preventive solid waste management practices such as separation of toxic wastes at their source, recycling of dangerous wastes, and waste minimization are preferred methods of managing landfill leachate quality. Moreover, siting of landfills in areas that are further from residential areas could minimize the risk of poisoning residents from contaminated groundwater.

Bulawayo is Zimbabwe's second largest city with a population of 700,000.^25^ All of the city's industrial and domestic solid waste is disposed at an unlined landfill, Richmond landfill. The landfill overlies a shallow unconfined aquifer, risking contamination of subsurface water in nearby residential areas. Bulawayo is heavily dependent on borehole water to augment its erratic piped water supplies, therefore contamination of sub-surface water could pose a serious public health concern.

The aim of this study was to monitor heavy metal release and assess contamination of sub-surface water from unlined Richmond landfill as a model of the environmental and public health hazards posed by sub-standard landfills.

**Abbreviations**
*Cd*: Cadmium
*Cr*: Chromium
*Cu*: Copper
*Pb*: Lead

## Methods

### Study Site

The present study was carried out in Bulawayo, Zimbabwe. Bulawayo is characterized by a semi-arid climate, and underground water is an indispensable supplementary source of water for its residents. All of the domestic and industrial solid waste generated in Bulawayo is currently being disposed of at Richmond landfill, which has been in use since 1989. The landfill covers an area approximately 206,208 m^2^ in size. It is located on a former gravel excavation site and is characterized by highly porous gravel soils. The landfill has three leachate ponds (P1, P2, and P3) which collect leachate from the landfill through underground drains. The leachate that collects in the ponds is neither treated nor pumped for safe disposal, but alternates cycles of accumulation (during rainy seasons) and natural evaporation (during dry, hot seasons). This creates a scenario where concentrations of contaminants in the leachate ponds increase over time. The landfill and leachate ponds are not membrane lined, except for a base layer of compacted clay, to minimize leachate seepage into the sub-surface. The porous soils beneath the landfill mean that breakthrough leachate can easily permeate into the sub-surface.

**Figure 1:**
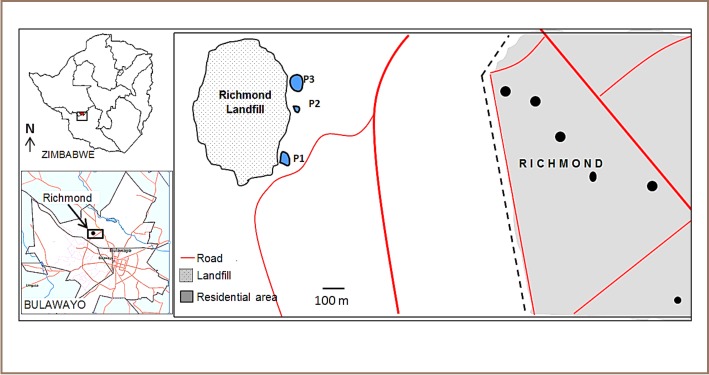
Map of Bulawayo indicating the location of Richmond Landfill, the three leachate ponds (P1, P2, and P3) and Richmond Suburb, east of the landfill. The location of the sampled boreholes (B1–B6) is indicated by black dots

**Figure 2:**
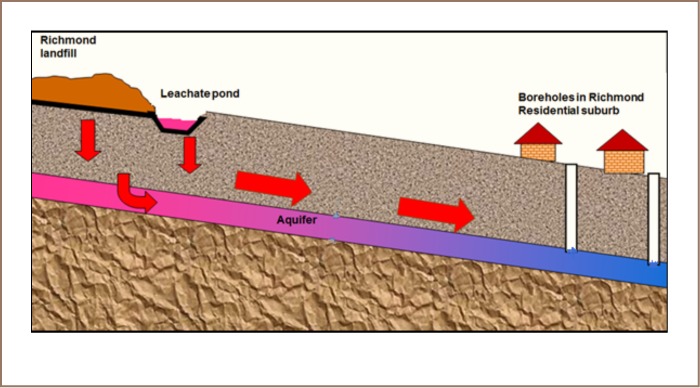
Conceptualized lateral view of Richmond Landfill and Richmond Suburb. The diagram indicates possible ways in which leachate from the aquifer could contaminate underground water (arrows) of Matsheumhlope Aquifer, risking the health of residents of Richmond Suburb who reside in the vicinity of the landfill

The topography and geology of the area surrounding the landfill and the nearby residential areas has been previously well characterized.^26^,^27^ The landfill overlies a shallow aquifer, Matsheumhlope aquifer, that has an average thickness of 40 m, and as a result, there is a high risk of groundwater contamination from the landfill.^27^,^28^ The aquifer has an effective porosity of 0.05, and hydraulic conductivity is 0.55 m/day, therefore contaminants from the landfill have the potential to migrate downgradient of the landfill.^28^ Groundwater flows around the landfill are known to follow the surface topography, therefore groundwater from the landfill flows towards the downgradient residential areas.^26^ Richmond residential suburb is situated within 800 m downgradient of the landfill at a gradient of 0.004 relative to the landfill.^26^ This represents a potential public health risk for residents of Richmond suburb who utilize groundwater from the aquifer for drinking. Richmond suburb and the area surrounding the landfill are generally composed of sandy loam soils. Figure 1 shows a map of Bulawayo and Richmond landfill. We hypothesized that the landfill leachate could be contaminating underground water and the pollutants could be reaching the Richmond suburb, as depicted in Figure 2.

Over the years, the landfill has attracted an informal settlement of a sizable population of scavengers who make their livelihoods selecting and selling recyclable and re-usable materials which include parts of electronic devices, and aluminum and copper, among other valuable wastes.

### Materials

All reagents for metal analysis were of analytical grade. These include nitric acid, hydrogen chloride, perchloric acid (Merck, Germany) and high purity metal standards (Fluka® Analytical, Sigma-Aldrich, Germany). As a quality control measure, all glassware that was used for handling and preparing samples for metal analysis was cleaned by soaking and rinsing in acidified distilled deionized water (1% nitric acid).

### Characterization of Solid Waste

The study was carried out soon after the rainy summer season, in the month of March. To characterize solid waste at the landfill, we made 2 M × 2 M transects (n=5) on top of the spread, compacted waste using strings and pegs. In each transect, we observed and categorized solid waste into soft plastics, hard plastics, paper, metals, electronic waste, organic waste and other, as described in Table 1. Each category was expressed as a percentage (volume) of total waste in each transect. The composition of each type of waste was the mean composition of the 5 transects.

**Table 1:**
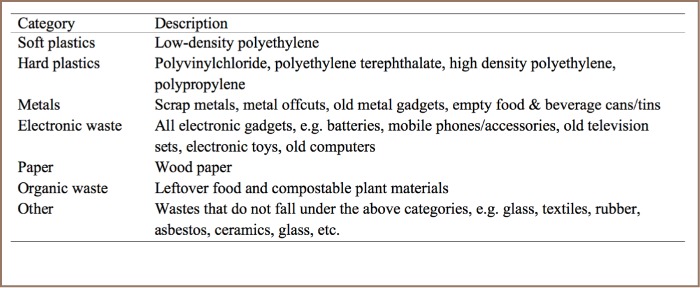
Categories of Solid Waste

### Sample Collection and Preparation

Landfill leachate was sampled from all leachate ponds (P1, P2, and P3) at the landfill using clean glass bottles. Leachate pH was recorded on site using a calibrated portable pH meter (Hanna, HI 98191). Three samples of soil (S1-S3) were collected at the foot, around the edges of the landfill from a depth of 5–15 cm. Another soil sample was collected at approximately 300 meters upgradient from the landfill and was used as the control soil sample to determine background levels of the metals. We identified two plant species, pigweed (Amaranthus hybridus) and jimsonweed (Datura stramonium), that were naturally flourishing at the foot of the landfill slope and assessed their metal uptake as indicators of pollution and metal bioavailability. Three mature plants of each of the two plant species were sampled by uprooting.

Water samples were collected from privately owned boreholes (B1–B6) in the nearby Richmond suburb to determine possible pollution of groundwater from leaching processes. The boreholes, B1–B6, which had depths ranging 13–25 m, were at increasing distances of 800, 970, 1200, 1310, 1600 and 2135 meters downgradient from the landfill. We were not able to get a “control” borehole within reasonable distance upgradient of the landfill, partly because there are no residential areas upgradient of the landfill.

### Metal Analysis

Soil samples were air dried until they reached a constant weight before pH and metal analysis. Soil for pH analysis was sieved through a 2 mm mesh, mixed with 0.01 M calcium chloride (CaCl_2_) to form a 1:2 (w/v) soil: CaCl_2_ solution slurry, mixed for 1 hour, and pH was measured using a calibrated glass electrode pH meter (Hanna, HI 98191). Soil for metal analysis was sieved through a fine (1-mm) mesh sieve to remove coarse soil particles. Plant samples were air dried. Drying continued until the samples attained constant mass and were pulverized using a porcelain mortar. Sieved soils and pulverized plant samples (1 g) were digested in 20 mL aquaregia (25% nitric acid: 75% hydrogen chloride v/v) for 24 hours. Each digested sample was heated (50°C–190°C) on a block digester to evaporate to near dryness. After cooling, 5 mL of perchloric acid was added and further heated at 70°C to near dryness. The final residue was transferred into a 50-mL volumetric flask and topped up to volume with deionized distilled water. The samples were filtered through ashless filter paper (Whatman®, International Ltd) before analysis. Landfill leachate and borehole water samples were filtered through ashless filter paper (Whatman®, International Ltd) to remove particulate matter and acidified with nitric acid to pH 2.0 before analysis. The filtered samples were analyzed for the metals Pb, Cd, Cr and copper (Cu) using atomic absorption spectrometry (Perkin Elmer, model 3110). The detection limits for Cu, Pb, Cr and Cd were 0.01 ppm. High purity metal standards endorsed by the National Institute of Standards and Technology (NIST) (Fluka® Analytical, Sigma-Aldrich, Germany) were used for instrument calibration and accuracy checks after analyzing every five samples. For quality assurance, we used standard soil and plant reference materials (NIST) for the selected metals.

Metal levels in soil and plants were expressed as mg Kg^−1^ dry weight, while metal levels in water were expressed as mg L^−1^. The levels of metal contamination of landfill soils (LS) in relation to background levels of control (CS) were expressed as contamination factor (CF), calculated as shown in Equation 1:

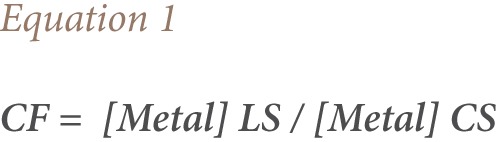



## Results

### Characterization of Solid Waste at Richmond Landfill

**Figure 3:**
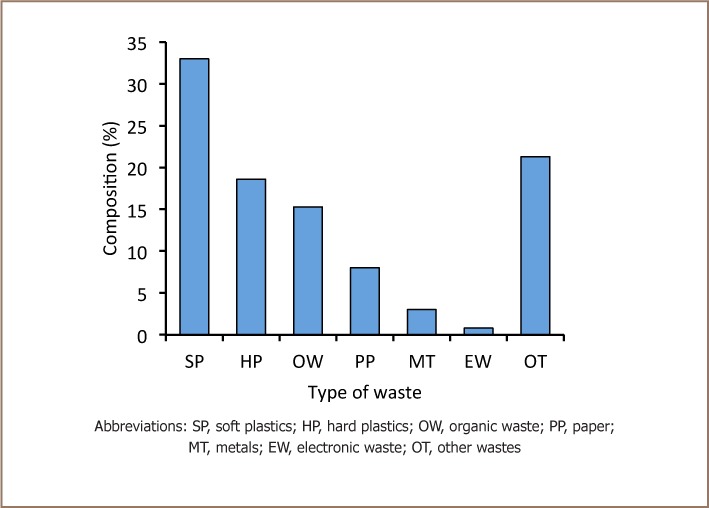
Composition (v/v) of solid waste at Richmond Landfill

Figure 3 shows the composition of various waste streams at the landfill. Major waste streams from Bulawayo consisted of soft plastics (33%), hard plastics (19%), organic compostable waste (15%), paper (8%), metallic wastes (3%) and electronic waste (0.8%).

### Metal Levels in Landfill Leachate, Soil and Plants

The mean landfill soil pH was 6.5±0.14 and mean pH of landfill leachate was 7.6±0.2. Table 2 and Table 3 show metal levels in landfill leachate and in landfill soil, respectively. All metals were present in leachate, soil, and in plants that were growing at the landfill. As expected, metal levels were much higher in soil. The contamination factors of landfill soil are shown in Table 4. Table 5 shows the levels of metals in roots and shoots of pigweed and jimson weed and the metal levels of surrounding soil from the rhizosphere.

**Table 2:**
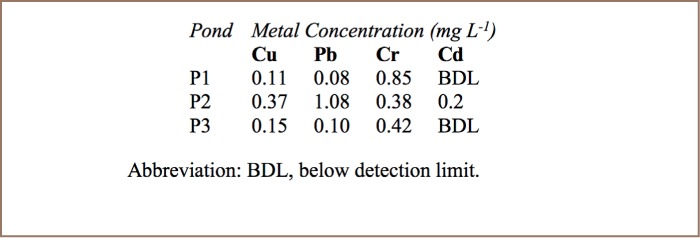
Metal Concentrations in Leachate From Richmond Landfill

**Table 3:**
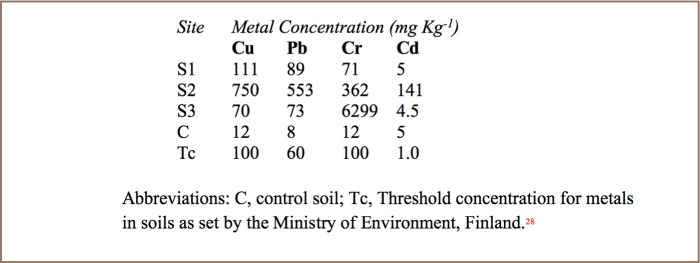
Mean Metal Concentrations in Soil Around Richmond Landfill

**Table 4:**
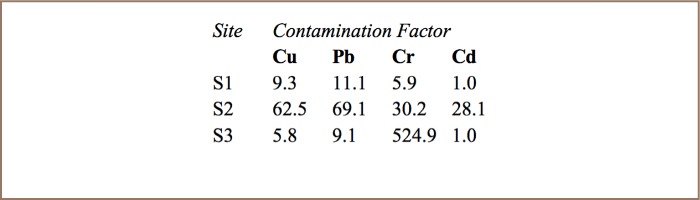
Contamination Factors of Soil at Richmond Landfill

**Table 5:**
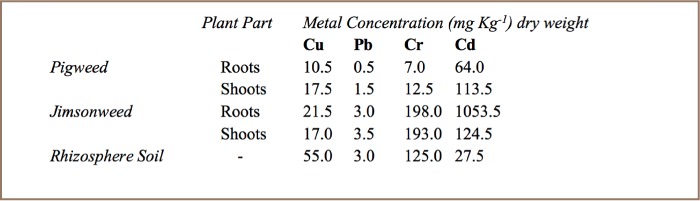
Heavy Metal Levels (mean) in Pigweed and Jimsonweed at Richmond Landfill

### Metal Levels in Borehole Water

**Figure 4:**
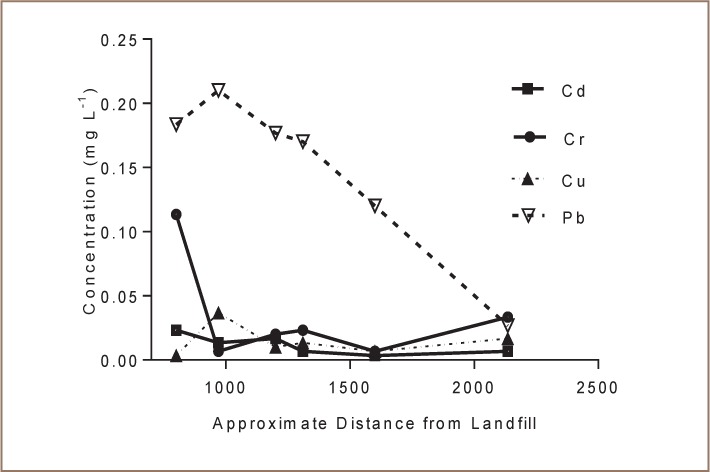
Concentrations of various metals in borehole water against distance from the landfill

**Figure 5:**
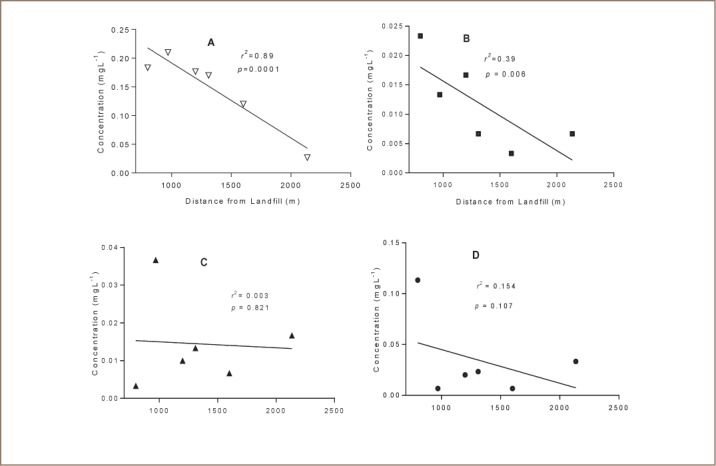
Linear regression analysis of concentrations of lead (A), cadmium (B), copper (C) and chromium (D) against distance from the landfill. The levels of Pb (r^2^=0.89; p=0001) and Cd (r^2^=0.39; p=0.006) were significantly correlated to distance from the landfill.

Concentrations of various metals in borehole water sampled at increasing distance from the landfill are shown in Figure 4 and Table 6. There was a significant linear relationship between distance from the landfill and Pb and Cd concentrations (*Figure 5*).

**Table 6:**
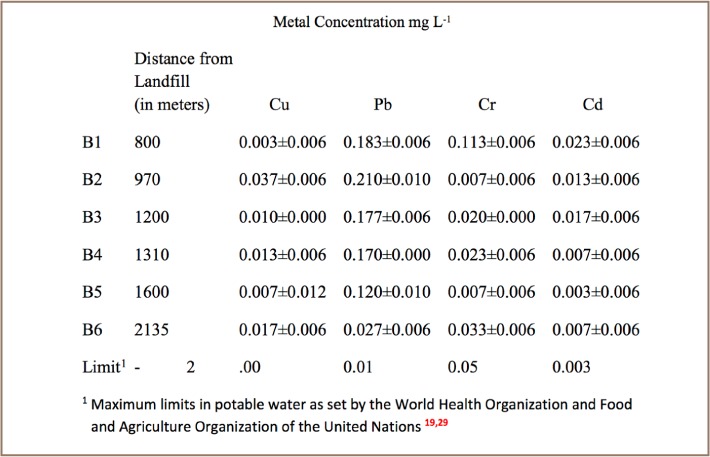
Metal Levels (mean± standard deviation) in Water from Boreholes in Richmond Suburb

## Discussion

Waste composition is a principal determinant of leachate quality.^22^ Metals, metallic wastes and electronic wastes contribute a significant proportion of heavy metals in landfill leachates. Bulawayo, similar to other cities in developing countries, does not separate toxic waste for recycling or safe disposal, therefore landfilled waste is varied, and includes metals and electronic wastes (*Figure 3*). There were significant quantities of metals (3%) and electronic waste (0.8%) at Richmond landfill, which were the likely sources of heavy metal contamination. Separation of metals and electronic wastes at the source for recycling or safe disposal are possible long-term measures to minimize leachate metal content and underground water contamination. The presence of significant quantities of recyclable waste such as paper and compostable organic matter presents the opportunity for Bulawayo to consider setting up waste recycling facilities. The waste composition obtained in this study could vary slightly from the composition of waste produced in Bulawayo. This is because some waste is systematically selected for re-use and recycling by scavengers. Scavengers target high value wastes such as aluminum, copper and reusable and valuable electronic devices. Scavenging can have positive effects of reducing landfilled wastes, particularly sources of heavy metals at landfills. The effect of the practice of scavenging on heavy metal contamination at Richmond landfill is unknown.

All assessed metals were detected in landfill soil, landfill leachate and in plants that were naturally flourishing at the landfill. Metal levels were clearly much higher in soil than in leachate since metals are generally less mobile and adsorb onto soils, especially at near neutral to alkaline soil pH ranges. The average soil pH was 6.5, which is near neutral and generally favors metal precipitation and adsorption onto soils and organic matter.

Although dissolved heavy metals (ions) precipitate out of solution when their pH is raised to a given point (pH 7.5–11), the optimum pH for precipitation varies, depending on the type of metal (e.g. Cr = pH 7.5; Cu = pH 8.1; Pb = pH 10.0; Cd = pH 11.0).^30–32^ A pH of high precipitation of one metal may be a pH of high solubility for another metal. At the pH of soils (pH 6.5), leachate (pH 7.6), and groundwater (pH 7.8), Cr and Cu are highly insoluble compared to Pb and Cd. This may partly explain why underground water and leachate have low levels of Cu and Cr, yet they occur at high concentrations in landfill soil. We did not analyze surface soil samples at sites of increasing distances downgradient of the landfill. However, we hypothesize that soil contamination from Cr and Cu is likely to be minimal and confined to the landfill site. The near neutral soil and leachate pH suggests that there is less likely to be any danger of Cr and Cu contamination from seepage, however at the pH of soil (pH 6.5) and leachate (pH 7.6), Pb and Cd have high solubility, therefore may contaminate the surrounding soils.

There was large variation in metal concentrations among the soil samples at the landfill (*Table 3*). This can be explained by the fact that the neutral pH of soils reduces the mobility of metals, making their concentrations in soils highly heterogeneous, and reflecting the metallic wastes at each soil sampling site. The leachate ponds P1–P3 receive leachate from three different “cells” of the landfill, with P1 being the oldest and P3 the newest cell. There were large variations in metal levels (*Table 2*) among the three leachate ponds (P1–P3), with P2 having higher concentrations of Cu, Pb and Cd than P1 and P3. The variations in leachate quality are largely determined by the characteristics of each cell in terms of waste composition, age of the cell and depth of waste, moisture content and available oxygen, all of which affect metal content in leachates.^33^

Leachate pH, the type of landfill lining and the geology of the sub-surface are important factors that determine the mobility of metals into the sub-surface. As noted earlier, the pH of landfill leachate (pH 7.6) favors precipitation of Cr and Cu from the aqueous phase into bottom sediments where they become less mobile.^34^,^35^ Unless there is a significant drop in leachate pH, Cr and Cu present minimal risk of groundwater contamination.

The leachate in the ponds is neither pumped nor treated, but goes through alternating cycles of accumulation and natural evaporation. Thus, pond sediments will gradually accumulate excessive levels of metals which can present a potential source of pollution in the event of changes in leachate pH into more acidic pH. Although the possibility of acidic pH values is minimized in aging landfills, natural phenomena such as acid rain and changes in waste composition can cause temporary acidification of leachate and increase the mobility and toxicity of sediment-bound metals.^36^ The practice of treating or pumping leachate for safe disposal reduces the risk of contaminating the environment from leachate overflows, particularly during flooding.

Levels of metals in borehole water from the adjacent residential area indicated some level of contamination. Borehole water from the nearby residential suburb had elevated levels of Pb (mean = 0.15 ppm) and Cd (mean = 0.01 ppm), which were correlated (p<0.01) to distance from the landfill (*Figure 5*). The concentration of Pb and Cd increased with decreasing distance from the landfill (*Figure 5*), suggesting that the landfill might be responsible for the contamination. Our conclusions are also based on previous studies that indicated that groundwater flows downgradient from the landfill towards the Richmond residential suburb.^26^

Geological characteristics of the subsurface also affect groundwater metal contamination.^37^ The aquifer is prone to pollution from the landfill due to its shallow depth and is characterized by fractured crystalline metabasalt formations, therefore pollutants can easily flow into groundwater.^27^ Our results are in agreement with previous studies by Kubare et al., who similarly reported lead migration from the landfill, with average lead levels of 0.21 ppm in Richmond area boreholes.^26^

Groundwater contamination from landfills has been a widely reported environmental menace caused by the leaching of solid wastes (e.g. Longe and Enekwechi, 2007; Mor et al., 2006; Nagarajan et al., 2012).^38–40^ The contamination of groundwater indicates that compacted clay is not a reliable lining material as some leachate seeps into the subsurface, making landfills serious sources of toxic pollutants.

The levels of Pb (0.03–0.21 mg Lz) and Cd (0.01–0.02 mg L^−1^) in groundwater were above the World Health Organization maximum potable water limits of 0.01 ppm and 0.003 ppm, respectively, making the water unsuitable for drinking.^19^ The adverse human health effects of lead and cadmium include neurological disorders and kidney and brain damage.^19^,^20^ To avoid adverse public health effects, an aerial assessment of the extent of metal contamination is needed, as well as raising public awareness of the hazards of drinking contaminated groundwater. The presence of Cr and Cu in landfill leachate and in soil and their absence in all of the groundwater samples can be attributed to their precipitation in the alkaline leachate and their affinity for adsorption to clay.

## Conclusions

This study indicates that there is potential pollution of groundwater from Pb and Cd that is migrating from Richmond landfill and we recommend an ensemble of remedial measures to curb the public health threat posed by the landfill, including separating metallic and electronic wastes for safe disposal and improved leachate management. The results of this study underscore the need for safer waste management practices to ensure environmental and public safety, particularly in developing countries such as Zimbabwe.
